# CSF2 Impairs Nrf2 Signaling through the Akt/Mtor Pathway in the Development of Bladder Cancer

**DOI:** 10.7150/jca.94343

**Published:** 2024-04-19

**Authors:** Xi Yu, Shenglan Li, Shuai Ke, Chenglin Ye, Qinghua Wang, Huaxin Wang, Lei Wang

**Affiliations:** 1 Department of Anesthesiology, Renmin Hospital of Wuhan University, Wuhan, 430060, Hubei, China.; 2 Department of Radiography, Renmin Hospital of Wuhan University, Wuhan, 430060, Hubei, China.; 3 Department of Urology, Renmin Hospital of Wuhan University, Wuhan, 430060, Hubei, China.

**Keywords:** bladder cancer, CSF2, AKT/mTOR pathway, Nrf2, EMT

## Abstract

Bladder Cancer (BCa) is one of the most common cancers of the urinary system. Colony-stimulating factor 2 (CSF2) is involved in many cancers, but not BCa. We investigated the effect of CSF2 on BCa in this study and the underlying molecular mechanisms. CSF2 mRNA levels in BCa were analyzed using the Cancer Genome Atlas (TCGA) database. Western blot was conducted to verify CSF2 expression in BCa tissue samples and cell lines. The effect of CSF2 on the growth of BCa cells was assessed by CCK8 and colony formation. To determine the migration and invasion capabilities of BCa cells, transwell analysis and wound healing assays were conducted. Next, western blot was used to explore the underlying mechanism. In the end, a xenografted BCa mouse model was established to examine the effects of CSF2 on tumorigenesis *in vivo*. Results showed that CSF2 mRNA was upregulated in BCa samples. Knocking down CSF2 significantly inhibited the proliferation and tumorigenesis of BCa cells *in vitro* and *in vivo*. Mechanism analysis revealed that CSF2 knockdown inhibited the proliferation and invasion of BCa cells via AKT/mTOR signaling. Based on these results, CSF2 promotes the proliferation and tumorigenesis of BCa.

## Introduction

There are estimated to be more than 16,000 deaths and 83,000 new cases of bladder cancer (BCa) in the United States in 2023 1. Cigarette smoking, occupational exposure, inflammation, and chemotherapy are thought to contribute to the high male-female ratio of new cases 2. In the case of BCa, there are a number of treatment options available. The options are recommended in urology guidelines, including radical cystectomy, chemotherapy, and intravenous immunotherapy with Bacteria Calmette Guerin (BCG) 3, 4. After treatment, the quality of life of BCa patients decreases dramatically and most BCa patients are unable to avoid tumor progression and recurrence. Tumor recurrence and distant metastases are the leading causes of death in BCa patients, with over 90% of BCa patients dying from distant metastases. In this regard, the development of improved treatments for BCa is a worthwhile endeavor.

In addition to stimulating stem cells to produce granulocytes and monocytes, colony-stimulating factor 2 (CSF2), also known as macrophage colony-stimulating factor 5. Activated T lymphocytes, fibroblasts, endothelial cells, and dendritic cells (DC) secrete CSF2 to promote dendritic cell recruitment, maturation, and function to induce protective immunity 6. CSF2 requires the ligand-specific subunit (GM-CSFR) receptor to activate intracellular molecular signalling pathways and thus promote cell growth, proliferation, and differentiation. Several recent studies have found that upregulation of CSF2 in the solid tumour microenvironment leads to poor prognosis in patients, which may result from suppressed immune responses 7. In other studies, CSF2 has also been found to be closely associated with poor prognosis in some tumours 8. However, the relationship between CSF2 and BCa remains unclear.

There is evidence that Nrf2 is implicated in antioxidant and cytoprotective responses. A stable interaction between Nrf2 and Keap1 occurs in the cytoplasm under normal conditions. The Nrf2 protein translocates from the cytoplasm to the nucleus when cells undergo oxidative stress and binds to Maf to form a heterodimer that further activates ARE-mediated downstream gene expression, thereby inducing proliferation and other phenotypic changes in tumour cells 9. Nrf2 has been shown to be highly activated in studies in BCa, where upregulation of Nrf2 leads to poor prognosis in BCa patients, and that overexpression in BCa cells accelerates tumour cell progression 10, 11.

In this study, the function of CSF2 in the development of BCa and further research was conducted on the molecular mechanisms by which CSF2 regulates Nrf2 in BCa.

## Materials and Methods

### Bioinformatics analysis

The public databases used in this paper are obtained through TCGA, GTEx, GEO and other public bioinformatics databases. The gene expression in TCGA and GTEx database comes from UCSC XENA, gene expression in GEO database, Methylation information is from Smartapp database: http://www.bioinfo-zs.com/smartapp/. All gene expressions in this study were standardized and log2 normalized. TCGA-BLCA contains 407 BCa samples and 19 BCa paracancerous tissues with paired adjacent samples; GTEx has 9 normal bladder mucosa tissues. GEO-GSE13507 contains 188 samples of BCa and 9 adjacent samples of BCa. The gene expression level of CSF2 in BCa and corresponding normal tissues was jointly mined by TCGA and GTEx, and then the ROC curve model of normal bladder tissues and BCa tissues was constructed by R language.

### Patients and tissue samples

A total of 52 BCa specimens and corresponding adjacent normal tissues were obtained from Renmin Hospital of Wuhan University from June 2021 to November 2022. Comprehensive clinical data were gathered and documented for each patient. All participating patients provided informed consent for tissue collection. The study was approved by the Ethics Committee of Renmin Hospital of Wuhan University. The inclusion and exclusion criteria are listed as follows: The inclusion criteria were: (1) postoperative pathology was BCa; (2) patients with complete clinical data. The exclusion criteria were: (1) immunosuppressed patients (for example, using HIV, chronic steroids); (2) patients suffering from other malignant tumors; (3) patients receiving chemotherapy or radiotherapy or other treatment before surgery.

### Antibodies

Proteintech (Wuhan, China) provided all of the primary antibodies: Secondary antibodies against goat anti-rabbit IgG and goat anti-mouse IgG were provided by Proteintech (Wuhan, China).

### Cells and culture

The human 5637 and T24 cell lines were purchased from ATCC. RPMI-1640 medium (HyClone, China) was used to maintain 5637 and T24 cells. They also received 1% penicillin G sodium/streptomycin sulfate along with 10% fetal bovine serum (FBS) (Gibco, Australia).

### Immunohistochemistry (IHC)

Then, similar to HE staining, dewaxing and rehydration were performed. Then, the tissue sections were boiled at 100°C for 15 min. Blocking with 3.0% hydrogen peroxide (H_2_O_2_) at room temperature for 10 min. After incubation with primary antibodies, the primary antibody was added to the tissue sections and incubated for one night at 4°C. Then, after adding the secondary antibody, 30 minutes of incubation were performed at room temperature the next day. At last, hematoxylin counterstained the sections after incubation with DAB chromogen.

### Western blot

Using RIPA buffer solution, 5637 and T24 cell lines were extracted of their total protein content. Discontinuous ultrasonic dispersion was used to split the samples. Lysates were centrifuged for 15 minutes at 4°C at 1 x 10^5^ g. The supernatant was detected with a bicinchoninic acid (BCA) assay. Protein samples were mixed with the loading buffer and denatured for 10 minutes at room temperature. Then, -20°C was used to store the protein samples. SDS-PAGE was used to separate 60 ug of protein from each sample and transfer it to NC membranes (Millipore, USA). Then, the membranes were immersed in 5% nonfat milk for 1 hour at room temperature. Then, incubation with primary antibodies overnight was followed by 1 hour of incubation with secondary antibodies. Finally, as part of the protein development process, bands were detected using an appropriate development instrument.

### CCK-8 assay

T24 and 5637 cell lines were seeded in 96-well plates at approximately 3 x 10^3^ cells/well and cultured for 24 h with different concentrations of brusatol. For each experiment, 10 µl of CCK-8 reagent (CK04, Dojindo, Japan) was added to each well and then cultured for 1 h. Absorption values were measured at 450 nm. The experiment was repeated three times.

### Colony formation assay

T24 and 5637 cell lines were seeded in 6-well plates at approximately 300 cells/well and grown for approximately 14 days. The colonies were counted using a microscope after 4% paraformaldehyde (PFA) fixation and 0.1% crystal violet staining. The experiment was repeated three times.

### Transwell migration assay

We used a transwell chamber system (Corning, USA). In the upper chamber, which was precoated with Matrigel (BD Biosciences, USA), 5 x 10^4^ cells were suspended in serum-free medium. Medium containing 20% FBS was added to the lower chamber. After 48 h, cells on the other side of the membrane were fixed in 4% PFA for 15 min and stained with 0.1% crystal violet for 1 h. The experiment was repeated three times.

### Wound healing assay

A 6-well plate was seeded with cells and cultured so the bottom of the plate was completely covered with them. Using a sterile 200 uL plastic pipette, the cell layer was scratched and washed with phosphate buffer solution (PBS) twice. Cells were cultured with medium containing 1% FBS for 48 hours. Photographs were taken at each time point. The experiment was repeated three times.

### Cell apoptosis

Flow cytometry was used to analyze 5637 and T24 cell lines stained with Annexin-V FITC. Three repetitions of the experiment were conducted.

### TUNEL assay

A kit for detecting apoptosis was used to perform the TUNEL assay. To observe the results, an optical fluorescence microscope (Olympus) was used.

### Reverse transcription (RT)-PCR and Quantitative (q)PCR

Total RNA was extracted from T24 and 5637 cell lines. RNA was extracted with the TRI (Absin, China). Reverse transcriptase reactions were performed using a SuperScript First-strand Synthesis System (Thermo Scientific, the USA). The cDNA was stored in a -20 ˚C refrigerator for use. Real-time PCR reactions were performed with GAPDH as internal control and using a NovoStart were qPCR SuperMix Plus (Novoprotein). Pre-denaturation occurred at 95°C for 30s; then the PCR reaction stage was entered with the conditions being 95°C 5s to 60°C 30s, this stage cycled 40 times, and finally the lysis stage was entered. Gene levels were shown as fold change relative to control calculated by the 2^-ΔΔCT^ method. Primers used for qPCR were listed as follows:

CSF2: 5'-GGAGCATGTGAATGCCATCCAG-3', 5'-CTGGAGGTCAAACATTTCTGAGAT-3';

NRF2: 5'-CACATCCAGTCAGAAACCAGTGG-3', 5'- GGAATGTCTGCGCCAAAAGCTG -3';

GAPDH: 5'-GTCTCCTCTGACTTCAACAGCG-3', 5'- ACCACCCTGTTGCTGTAGCCAA-3'.

All experiments were conducted in triplicate and repeated three times.

### Cell transfection

The small interfering RNAs (siRNAs) used to downregulate CSF2 expression were purchased from Genechem (Shanghai, China). The full sequence of NRF2 was inserted into a lentiviral vector (Sangon Biotech, China). Then, SiRNAs and cDNA were transfected to T24 and 5637 cell lines respectively according to the manufactory's description. Infection efficiency was measured with Western blot and real-time PCR.

### Xenograft mouse model

Renmin Hospital Ethics Committee of Wuhan University approved the study. The animals used in the present study were performed in accordance with the Animal Welfare Act. Using a CSF2 knockdown lentivirus obtained from Genechem (Shanghai, China), T24 cells were transfected. Our xenograft mouse model was based on 4-week-old male nude specific pathogen-free mice (SPF). They were subcutaneously injected with 1.5 x 10^6^ CSF2 knockdown or negative control cells that were resuspended in PBS. Approximately four weeks later, the mice were sacrificed by sodium pentobarbital (100 mg/kg) and the tumors were dissected and stained with IHC.

### Statistical analysis

All values are presented as the mean ± Standard Deviation of the replicate samples. All experiments were repeated three times. The 22.0 SPSS software package was used for all statistical analyses.

## Results

### CSF2 upregulation in BCa tissue specimens and cells

**Figure 1:**
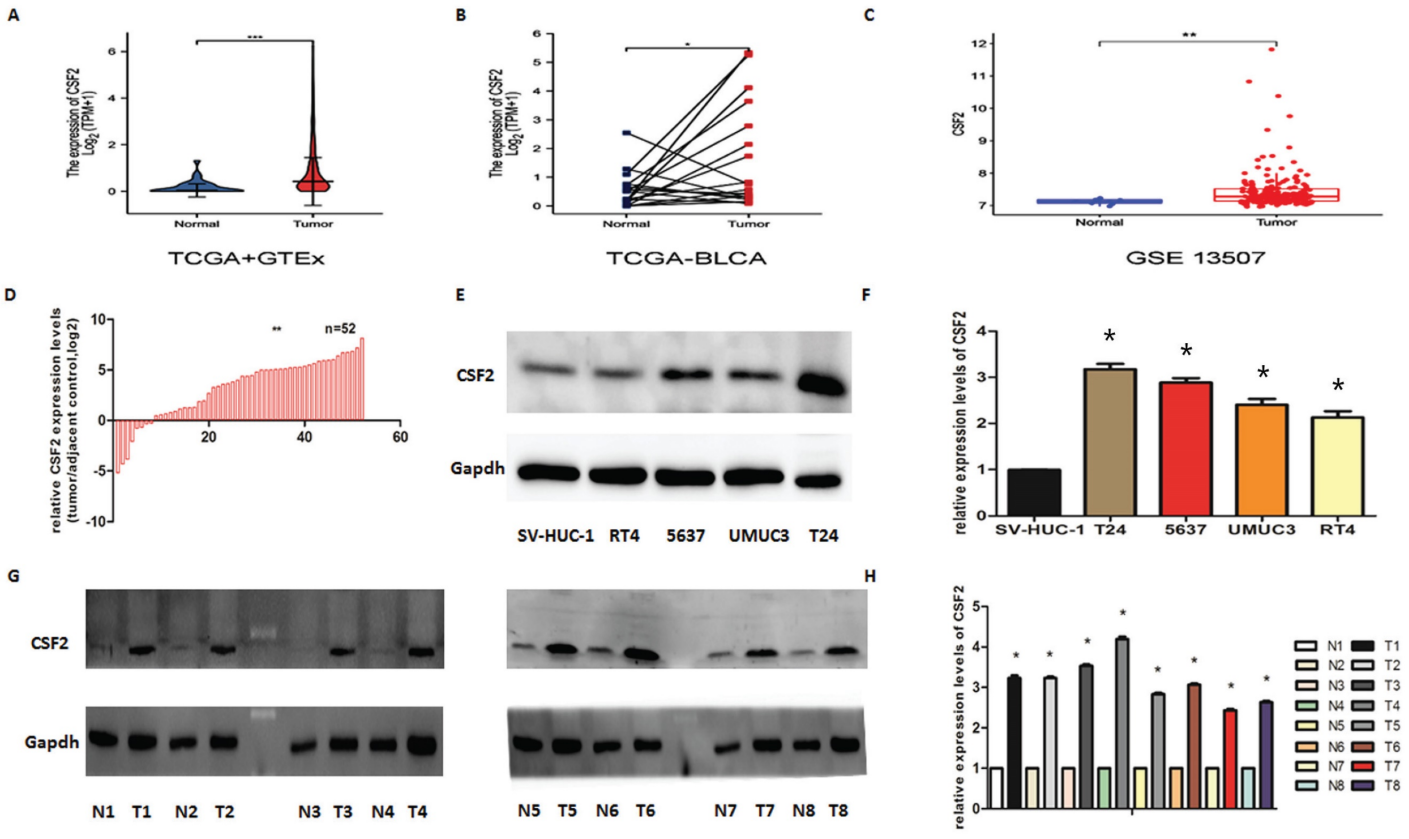
CSF2 is upregulated in BCa. (A) CSF2 expression in 28 normal tissues and 407 BCa tissues included in TCGA and GTEx. (B) CSF2 expression in 19 cases of BCa tissues and adjacent tissues included in TCGA. (C) CSF2 expression in 19 cases of BCa and adjacent tissues included in GEO. (D) CSF2 mRNA expression in BCa tissue samples was high than in normal tissue. (E) Western Blot showed that the protein level of CSF2 was upregulated in all of the four bladder cell lines compared to that in normal epithelial cells. (F) RT-qPCR analysis showed that the mRNA level of CSF2 was indeed upregulated in all of the four bladder cell lines compared to that in normal epithelial cells. (G/H) Western blot analysis revealed that the protein level of CSF2 were upregulated in malignant BCa tissues compared to that in adjacent normal tissues. Values are expressed as the mean ±SDs. *P < 0.05.

The CSF2 expression level in 28 normal tissues and 407 BCa tissues included in TCGA and GTEx was different (Figure 1A). The expression level of CSF2 in 19 cases of BCa tissues included in TCGA was different from that in paired adjacent tissues (Figure 1B). The CSF2 expression level in 19 cases of BCa included in GEO was different from that in the paired adjacent tissues (Figure 1C). The expression of CSF2 in BCa needed to be clarified, so we first examined it in BCa cell lines using WB and qRT-PCR. We first assessed CSF2 mRNA expression in BCa tissue samples compared to normal tissue samples. qRT-PCR showed that CSF2 mRNA was significantly higher in the BCa tissue specimens than in the normal tissue specimens (Figure 1D). Other results suggested that the protein expression (Figure 1E) and RNA content (Figure 1F) of CSF2 were significantly higher in RT4, 5637, UMUC3 and T24 relative to normal uroepithelial cells (SV-HUC-1), especially in T24 and 5637 cell lines. By extracting fresh tissue proteins for western blot assay, we found that compared to paracancerous tissues, BCa tissues contained significantly more CSF2 protein (Figure 1G/1H). The clinicopathological factors of the 52 patients are listed in Table 1. As shown in Table 1, the expression of CSF2 was not related to age, gender, smoking status, BMI, diabetes status, or tumor size but significantly correlated with T stage and histologic grade. CSF2 may be important in BCa, according to these results. From these data, we infer that CSF2 might contribute to the progression of BCa.

**Figure 2:**
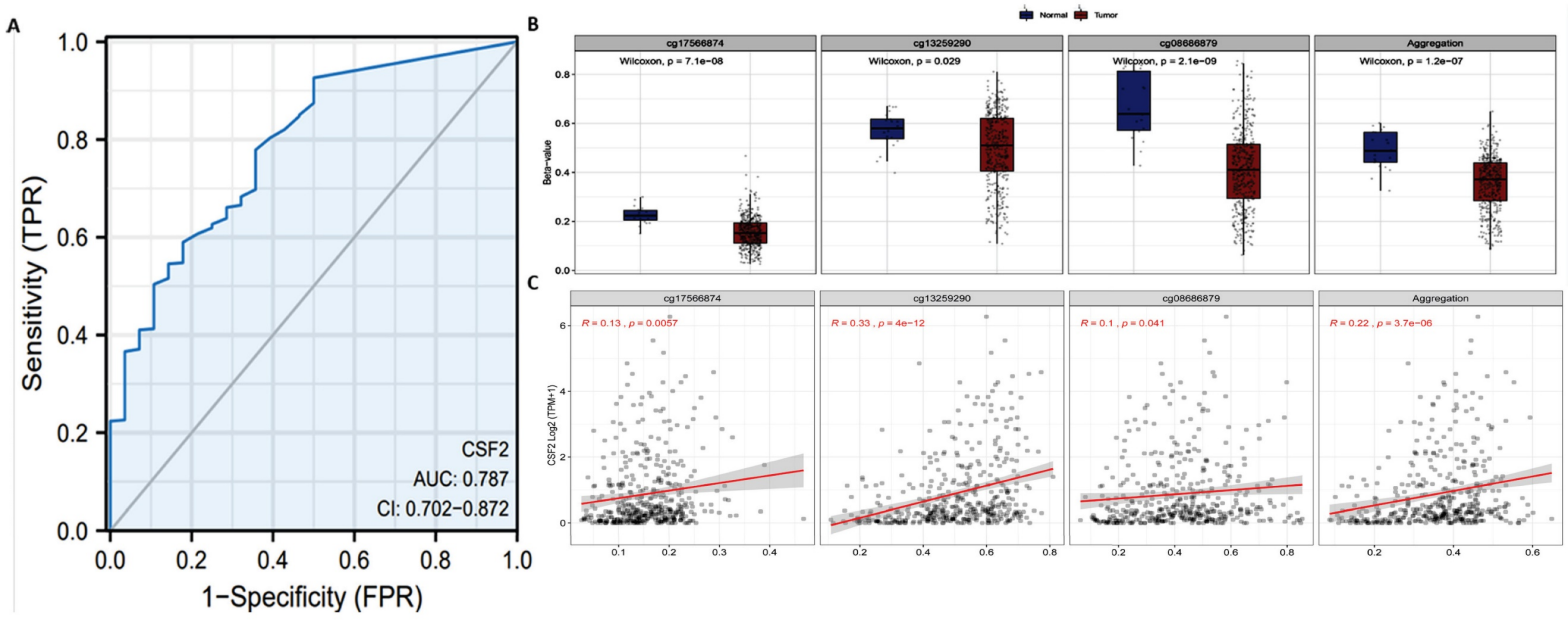
(A) ROC curve of CSF2 in adjacent tissues of BCa and normal tissues. (B) Using cg17566874, cg13259290 and cg08686879 as probes, the promoter methylation level of CSF2 gene in normal and tumor tissues was detected. (C) The probes of cg17566874, cg13259290, cg08686879 were negatively correlated with CSF2 gene expression.

The previous results have shown that the CSF2 gene level in BCa is significantly lower. In order to further evaluate the accuracy of CSF2 in BCa tissue and normal bladder tissue, TCGA and GTEx BCa data were extracted and ROC model validation was carried out. The results suggest that CSF2 has a degree of accuracy in distinguishing tumor tissue and normal tissue, with AUC reaching 0.787 (CI: 0.702-0.872) (Figure 2A). According to Smartapp database analysis, multiple probes (cg17566874, cg13259290, cg08686879) were used in the CSF2 promoter region of BCa tissue in prostate tumor tissue β (Figure 2B). The value is lower than that of normal tissue, and the expression of CSF2 follows β. The decrease of the value shows an upward trend (Figure 2C). We believe that the demethylation of the promoter region is one of the reasons for the up regulation of CSF2 expression.

### Proliferation, migration, and invasion of BCa cells are triggered by CSF2

**Figure 3:**
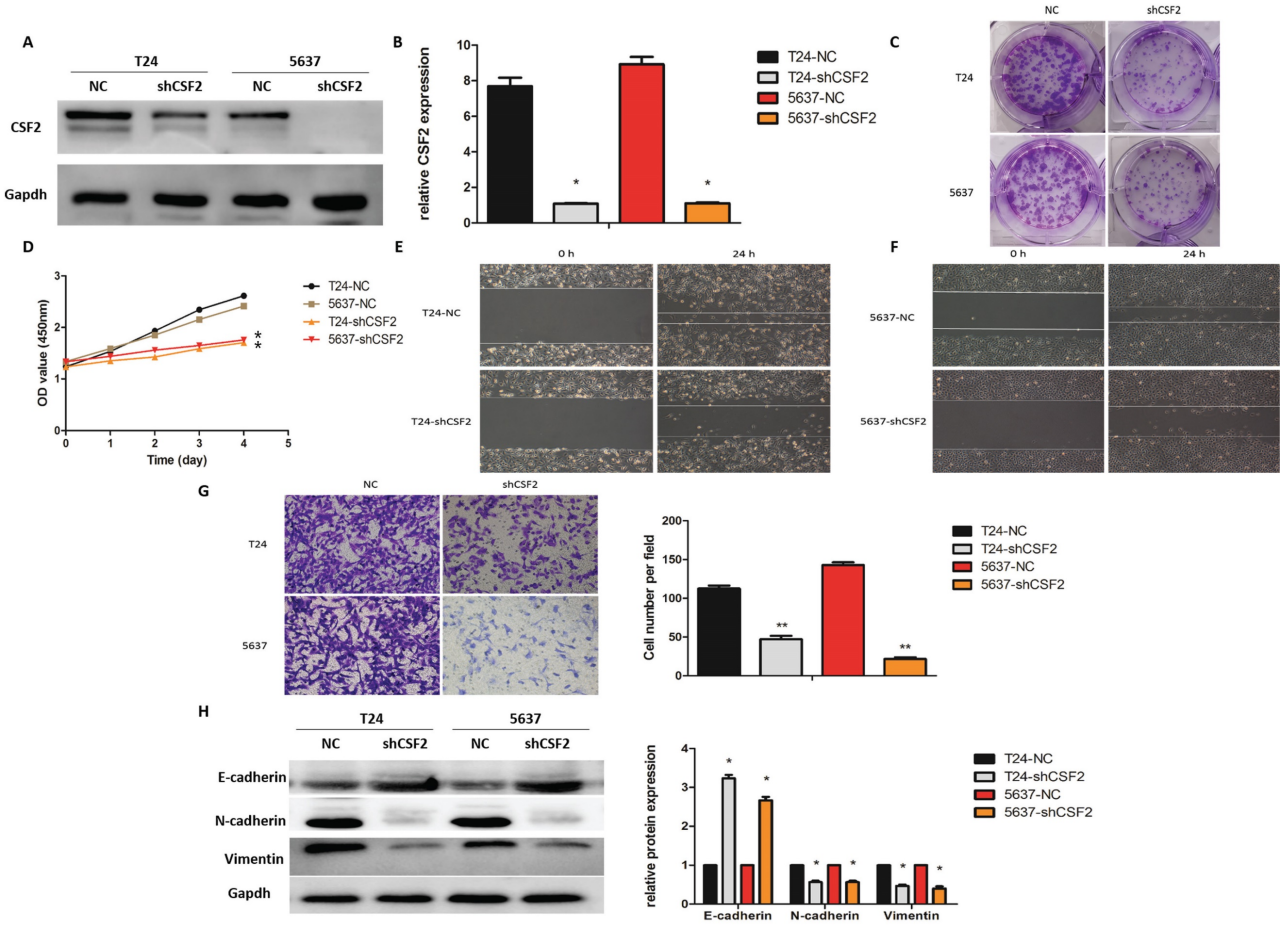
CSF2 regulates the proliferation, migration and invasion of BCa cells *in vitro*. We verified the knockdown efficiency of CSF2 in T24 and 5637 cell lines by western blot (A) and qRT-PCR (B) respectively. (C) The representative images from the colony formation assay showed that knockdown of CSF2 significantly decreased the mean colony numbers. (D) CCK-8 assays showed that knockdown of CSF2 significantly decreased the growth rate of T24 and 5637 cell lines. The Wound healing assays showed that knockdown of CSF2 decreased the migration ability of T24 (E) and 5637 (F) cell lines. (G) The transwell assay showed that knockdown of CSF2 decreased the invasion of T24 and 5637 cell lines (magnification x 200). (H) Western blot showed that EMT-associated markers such as E-cadherin was increased, whereas Vimentin and N-cadherin were decreased in CSF2 knockdown cells. Data are presented as the means ± SDs for 3 independent experiments. *P < 0.05 vs. the control group.

Next, in order to verify the role of CSF2 in the mid-stage of BCa, we first used shRNA to knock down CSF2 in BCa cell lines (T24 and 5637) using Western blot (Figure 3A) and qRT-PCR (Figure 3B) respectively. These results suggested that the CSF2 knockdown group of T24 and 5637 cell lines showed a decrease in cell proliferation ability compared to the control group (Figure 3C), and These results also suggested a significant decrease in cell viability of T24 and 5637 cell lines in the CCK-8 assay (Figure 3D). These results further suggested that the wound healing assay indicated that the CSF2 knockdown group of T24 and 5637 cell lines showed a decrease in cell migration (Figure 3E/3F). Additionally, the transwell assay showed a decrease in cell invasion ability (Figure 3G). To explore whether CSF2 regulated EMT-related markers, we performed western blot. These data suggested that E-cadherin levels increased, while Vimentin and N-cadherin expression levels decreased in the CSF2 knockdown group (Figure 3H). These results suggest that CSF2 knockdown inhibits the growth, migration and invasion ability of T24 and 5637cell lines.

### CSF2 inhibits apoptosis in BCa cells

**Figure 4:**
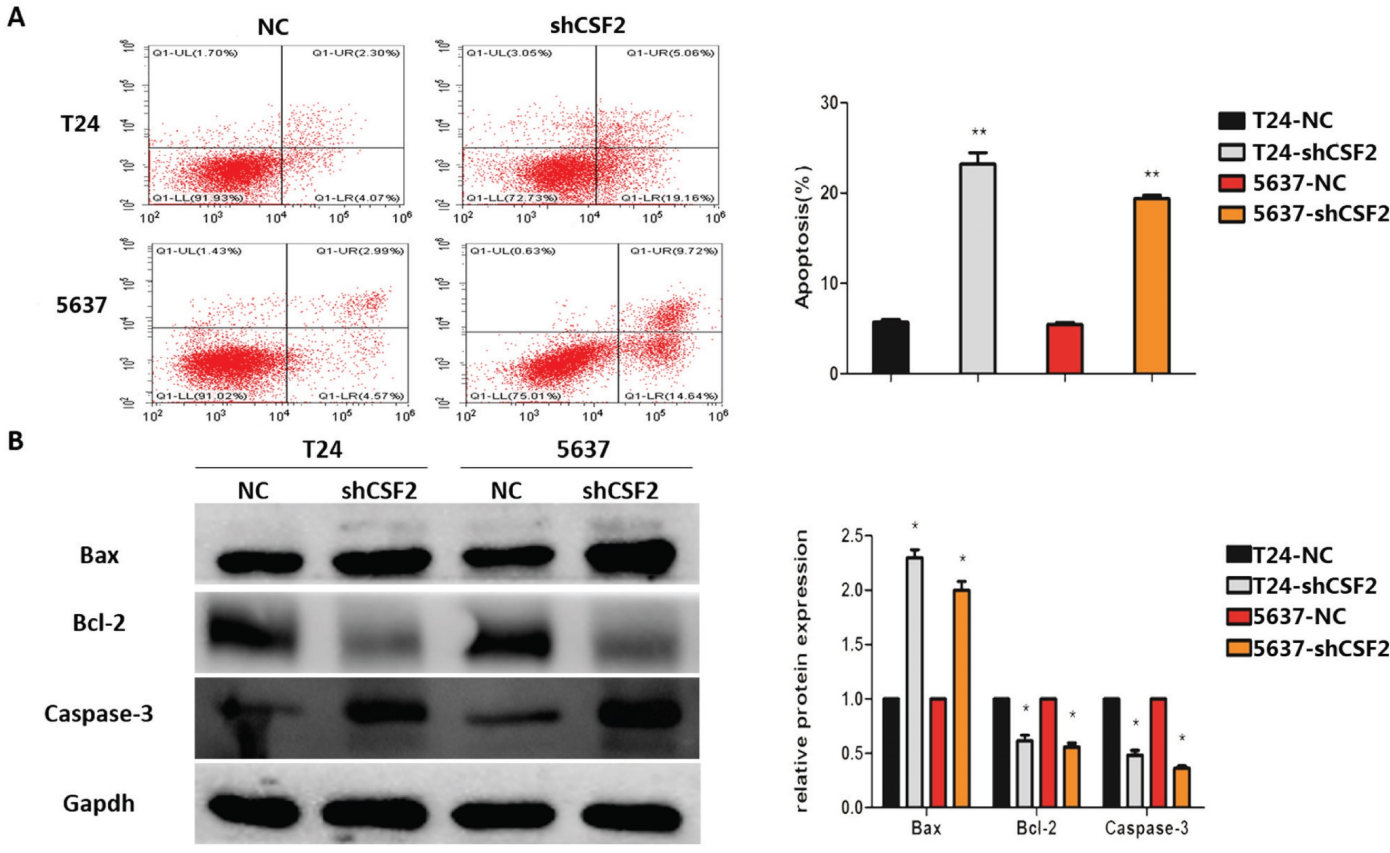
CSF2 inhibits apoptosis in BCa cells through activating caspase-3. (A) Flow cytometry assays showed that knockdown of CSF2 significantly decreased the apoptosis rate of T24 and 5637 western blots. Cell apoptosis was detected by flow cytometry using annexin V/PE staining. Representative flow cytometric images and statistical data are showed. (B) Western blot showed that apoptosis-associated markers such as Bcl-2 were decreased, whereas Bax and cleaved-caspase3 were increased in CSF2 knockdown cells. The data are presented as means ± SDs for 3 independent experiments. *P<0.05 compared with the control group.

Aiming to determine how CSF2 affects BCa cells to inhibit tumor growth, we use flow cytometry to determine whether it could induce apoptosis. As expected, compared to the control group, CSF2 knockdown group of T24 and 5637 cell lines exhibited a significantly higher rate of apoptosis (Figure 4A). Western blot was used to examine whether CSF2 suppresses apoptosis-associated markers in T24 and 5637 cell lines. Flow cytometry showed an increase in Caspase-3 cleavage and Bax expression, while Bcl-2 expression decreased (Figure 4B). All these results suggested that T24 and 5637 cell lines were more likely to undergo apoptosis when CSF2 was knocked out.

### CSF2 induces aggressive behavior in BCa cells by increasing Nrf2 expression

**Figure 5:**
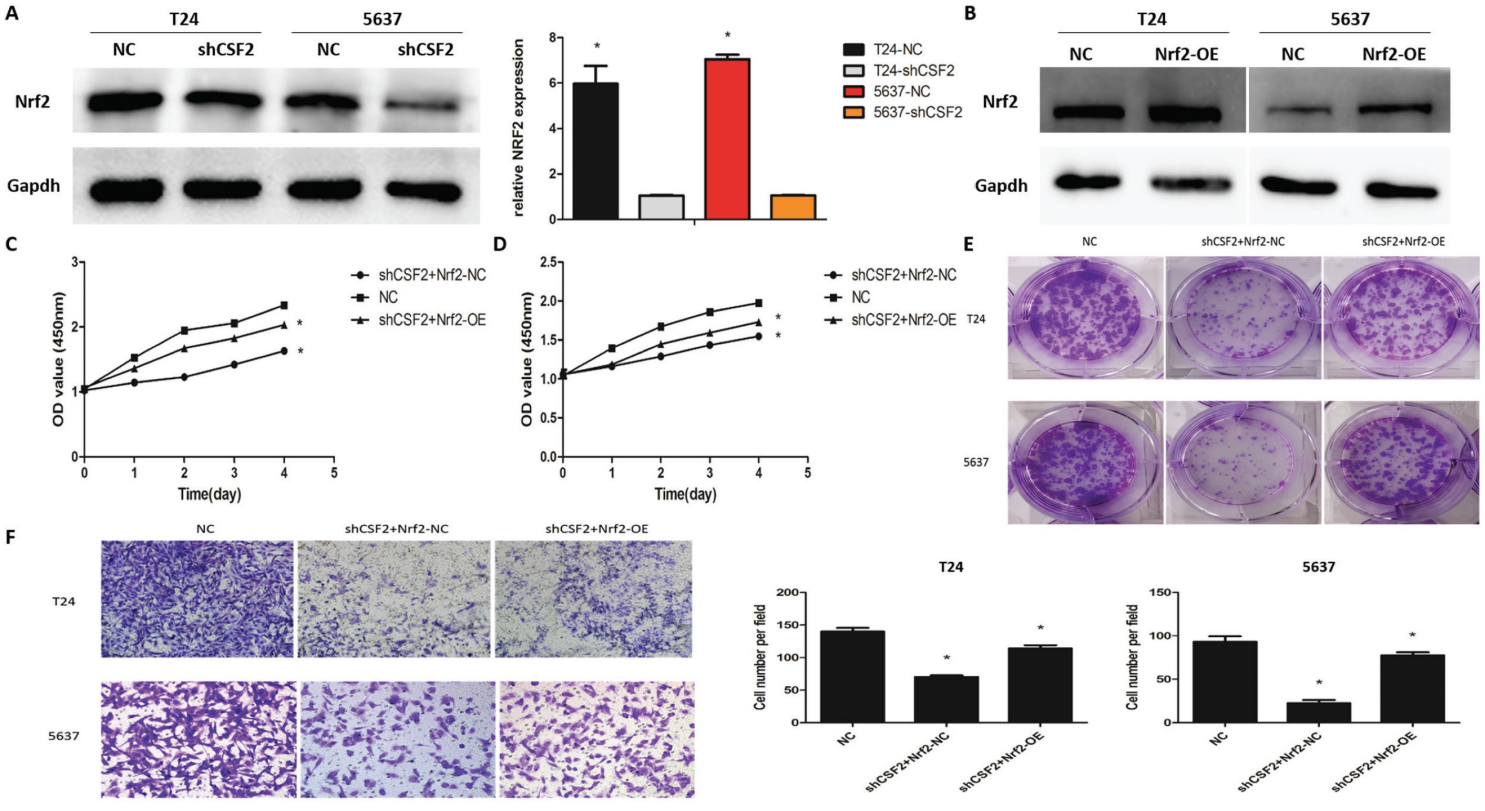
CSF2 prompts aggressive behaviors in BCa cells by increasing the expression of Nrf2. (A) The protein of Nrf2 was decreased in CSF2 knockdown T24 and 5637 cell lines. (B) The overexpression efficiency of Nrf2 (OE) in T24 and 5637 cell lines. The expression of Nrf2 were increased compared to negative control (NC). (C) CCK-8 assays showed that overexpression of Nrf2 reversed the inhibitory effect of CSF2 knockdown in T24 (C) and 5637 (D) cell lines. (E) Cell proliferation was assessed by the colony formation ability. The overexpression of Nrf2 reversed the inhibitory effect in CSF2 knockdown T24 and 5637 cell lines. (F) The transwell assay showed that overexpression of Nrf2 reversed the ability of invasion in CSF2 knockdown T24 and 5637 cell lines (magnification x 200). Data are presented as the means ± SDs for 3 independent experiments. *, P<0.05 vs. the control group.

To explore whether Nrf2 regulates cancer progression in T24 and 5637 cell lines, western blot was used to measure Nrf2 expression. These results suggested that the CSF2 knockdown group showed greatly reduced expression levels of Nrf2 (Figure 5A). T24 and 5637 cell lines overexpressing Nrf2 were constructed for further investigation (Figure 5B). The CCK-8 assay (Figure 5C, 4D), clone formation assay (Figure 5E), transwell assays (Figure 5F) revealed that boosting Nrf2 expression on T24 and 5637 cell lines partially counteracted the inhibitory effects of CSF2 knockdown. These results suggested that T24 and 5637 cell lines' behavior can be mediated by CSF2 through regulating Nrf2.

### CSF2 regulates Nrf2 via the Akt/Mtor pathway in BCa cells

**Figure 6:**
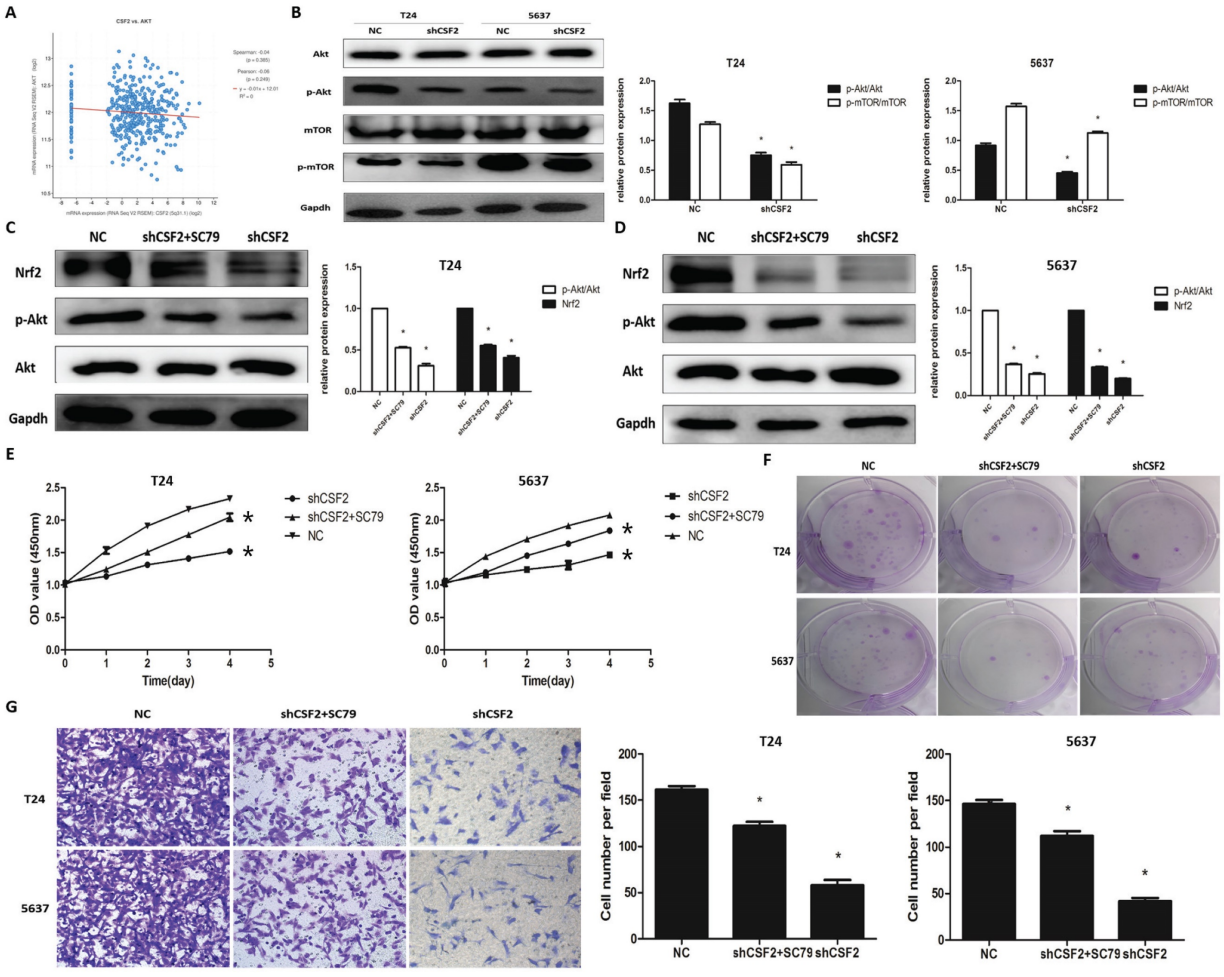
Akt/mTOR pathway is required for the regulation of Nrf2 by CSF2 in BCa cells. (A) There was a positive correlation between the expression of CSF2 and AKT pathway. Western blot revealed the levels of p-Akt and p-mTOR were significantly decreased in CSF2 knockdown T24 and 5637 cell lines (B). Western blot revealed that SC79 reversed the levels of Nrf2 and p-Akt in CSF2 knockdown T24 (C) and 5637 (D) cell lines. (E) CCK-8 assays showed that SC79 reversed the inhibitory effect of CSF2 knockdown in T24 and 5637 cell lines. (F) Cell proliferation was assessed by the colony formation ability. SC79 reversed the inhibitory effect in CSF2 knockdown T24 and 5637 cell lines. (G) The transwell assay showed that SC79 reversed the ability of invasion in CSF2 knockdown T24 and 5637 cell lines (magnification x 200). Data are presented as the means ± SDs for 3 independent experiments. *, P<0.05 vs. the control group.

The cBioPortal database was used to explore the specific mechanisms by which CSF2 influences BCa progression using KEGG pathway analysis, and the results suggest that CSF2 may regulate BCa progression through the signalling pathway in which AKT is located (Figure 6A). The cBioPortal database also provides preliminary confirmation that there is no clear correlation between CSF2 and AKT1 expression (S1) and that AKT's function is generated post-transcriptionally through phosphorylation modifications. We then performed Western blot experiments to verify changes in protein of p-Akt and Akt. Nrf2 is regulated by Akt/mTOR signaling in tumor cells 12, 13. In order to determine whether Nrf2 is regulated by the Akt/mTOR pathway, we examined the activation of this pathway in T24 and 5637 cell lines. In the end, the levels of p-Akt and p-mTOR were significantly decreased in CSF2 knockdown T24 and 5637 cell lines (Figure 6B). Subsequently, we use Akt agonist SC79. The cell biological activity assays were performed after the CSF2 knockdown T24 and 5637 cell lines were treated with SC79. Western blot revealed an increase in Nrf2 protein levels following SC79 treatment in CSF2 knockdown T24 (Figure 6C) and 5637 (Figure 6D) cell lines. In other experiments, SC79 treatment significantly reversed the inhibition of CSF2 knockdown T24 and 5637 cell lines (Figure 6E, 6F, 6G). Altogether, these results confirmed that CSF2 regulates Nrf2 in T24 and 5637 cell lines by activating Akt/mTOR.

### Knockdown of CSF2 inhibits the growth of BCa

**Figure 7:**
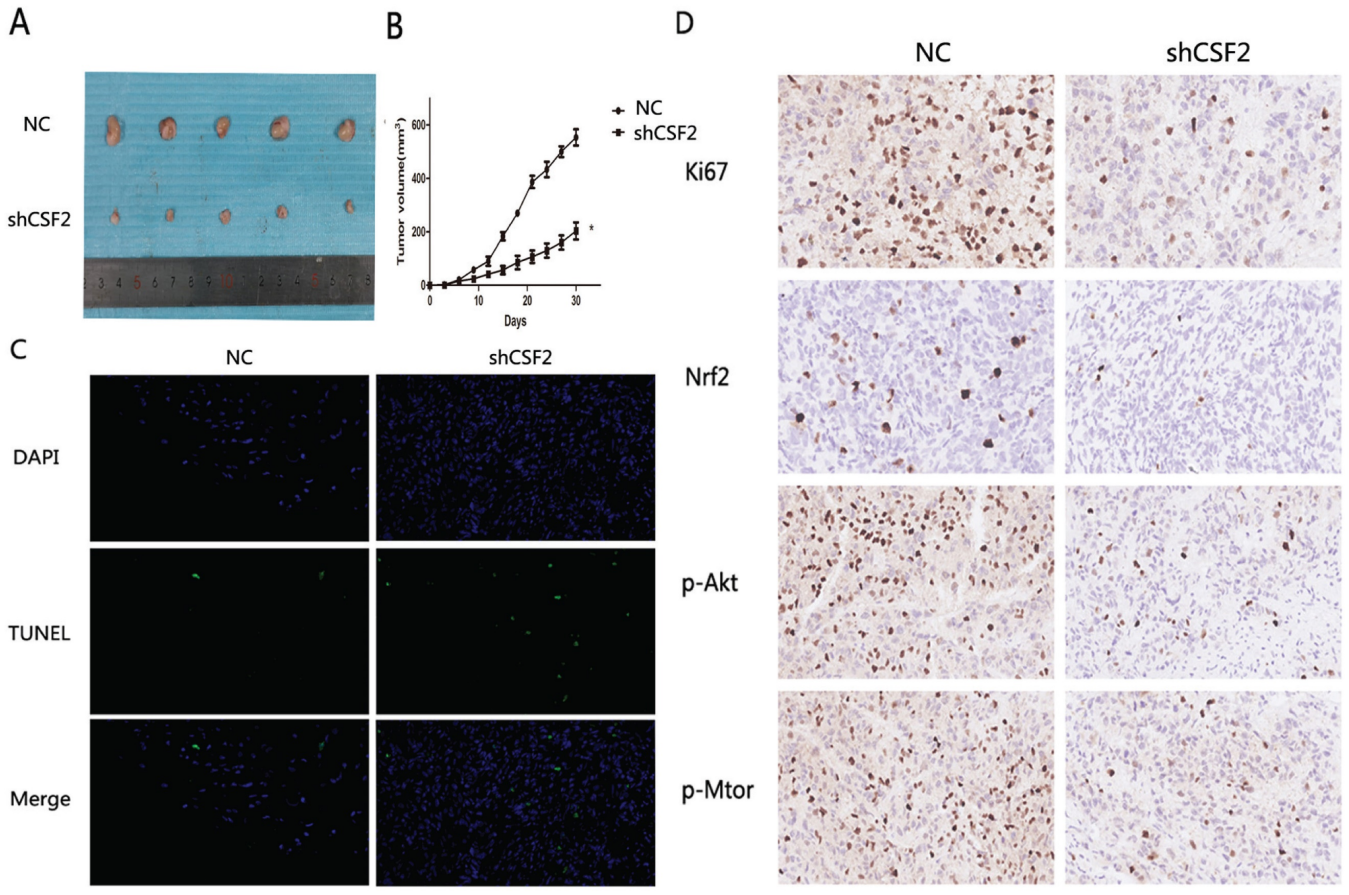
Knockdown of CSF2 significantly inhibits the growth of BCa *in vivo*. (A) Morphology of the subcutaneous implanted tumor. (B) Mean tumor volume at each time point. (C) A TUNEL assay was performed to detect the apoptotic cells in the tumor tissue (magnification x 200). (D) IHC was performed to detect the protein of Ki67, Nrf2, p-Akt, p-mTOR in the tumor tissue (magnification x 200). *P < 0.05 vs. the control. All the above data are the mean ± SD from an average of three experiments.

BCa cells were inhibited when CSF2 was knocked down *in vitro*. With a xenograft mouse model, we tested whether knocking down CSF2 inhibited BCa cell growth *in vivo*. The mice were all randomly divided into two groups and received T24-NC or T24-shCSF2 cells injection. A tumor size measurement was performed after each injection. At last, T24-shCSF2 injection mice had smaller tumors than their control group (Figure 7A). In T24-shCSF2 injection group, there was a noticeable difference between the tumor growth rate and the control group. (Figure 7B). Consequently, TUNEL staining levels were increased (Figure 7C), which indicated T24-shCSF2 induced apoptosis of tumor *in vivo*. Meanwhile, Ki-67 expression and Nrf2 levels were reduced by IHC analysis in the T24-shCSF2 injection group, as well as p-Akt and p-mTOR (Figure 7D). These data confirmed that knockdown of CSF2 significantly inhibits the growth of BCa *in vivo*.

## Discussion

The most common urogenital system tumor in adults is BCa 1. Because of BCa's aggressive nature and proliferation rate, recurrence is common despite surgery, radiotherapy, and chemotherapy 14. Therefore, the search for new therapeutic targets is of great importance for treating BCa. In this study, BCa tissues express more CSF2 than normal tissues, as opposed to normal tissues. As well, BCa with high CSF2 expression showed a high T stage and tumor grade. There was a superior survival rate in patients with low CSF2 expression than those with high CSF2 expression. BCa cells were inhibited *in vitro* and *in vivo* when CSF2 was knocked down. Consistently, we identified CSF2 could activate Nrf2 which played an important role in tumor biology. In addition, CSF2 activates the Akt/mTOR signaling pathway, up-regulating Nrf2 protein expression.

The upregulation of CSF2 in the solid tumor leads to poor prognosis in some tumors. Cai *et al.* reported that targeting CSF2/CSF2R for M2 macrophage reprogramming has been studied in clinical trials for cancer therapy 15. CSF2/CSF2R signaling plays an important role in macrophage polarization 15. Previous studies have demonstrated that the phosphorylation of STAT3/MYC by CSF2 regulates the phenotypic plasticity of small cell lung cancer 16. Lee *et al.* performed research about CSF2 in BCa 17. Patients with BCa and advanced disease status showed CSF2 was associated with poor clinical outcomes. The results suggested that prognosticators and potential therapeutic targets of BCa include CSF2. There is however a lack of knowledge about its role in BCa and the molecular mechanisms underlying it. Consistent with their findings, we found among BCa patients, high levels of CSF2 were associated with a high T stage and tumor grade. In addition, we revealed knockdown of CSF2 inhibited BCa cells *in vitro* and *in vivo*.

Firstly, we found that BCa cells were less capable of proliferating and surviving when CSF2 was knocked down. Regarding the mechanism underlying increasing tumor malignancy of CSF2, studies show that maybe connect to inducing apoptosis. Known as a programmed cell death, apoptosis occurs when cells die. A flow cytometry analysis confirmed the results in these reports that CSF2 knockdown induced apoptosis in BCa cells. BCa cells migrate and invade less when CSF2 is knocked down in another experiment. EMT of tumor cells may be the major cause of distant metastases from tumors, according to a growing number of studies 18-20. The majority of BCa deaths are caused by distant metastases of tumors. The loss of cell-to-cell connection and apicobasal polarity occurs during EMT, and the cells assume a fibroblast-like appearance, allowing metastatic spread 21, 22. E-cadherin is the key epithelial marker of EMT 23. CSF2 knockdown cells showed a significant decrease in migration in our study. Additionally, knocking down CSF2 inhibited BCa cells' EMT, attenuating BCa malignancy. It appears that CSF2 was an oncogene in BCa development, but further research is needed to confirm this.

An important transcription factor, Nrf2, plays a role in the survival of cancer cells. It has been shown that this protein can induce tumor-protective gene transcription, thereby promoting tumor growth and preventing cells from apoptosis 24, 25. As a result of the aryl hydrocarbon receptor (AhR), polycyclic aromatic hydrocarbons stimulate Nrf2. An AhR-binding nuclear translocator (Arnt) transactivates Nfe2l2 by transactivating its promoter that contains xenobiotic response element-like sequences. An activation of the Nrf2 pathway was caused by xenobiotic ligands induced by AhR 26. Human MCF10A mammary cells activate NFE2L2 in response to breast cancer susceptibility 1 (BRCA1) 27. A human mammary cell line, MCF10A, also activates NFE2L2 when breast cancer susceptibility 1 (BRCA1) is present 28. In addition to inhibiting EMT, Nrf2 also inhibited esophageal squamous cell carcinoma metastasis 29. This is consistent with our findings. CSF2 knockdown cells showed a marked decrease in Nrf2 expression. A significant reduction of inhibition on BCa cells was observed with Nrf2 overexpression. Since Nrf2 is ubiquitinated by E3 ligases and degraded by 26S proteasomes under normal conditions, we hypothesized that CSF2 knockdown would also affect Nrf2 30. During the deeply experiment, Nrf2 overexpression improved BCa's biological behavior, suggesting Nrf2 has a role to play in CSF2 regulating BCa.

There is no doubt that the Akt/mTOR pathway plays a significant role in various types of cancer. Tumor cells are more likely to proliferate and metastasize when Akt is activated, since it inhibits its downstream molecules. Inhibition of downstream targets is accomplished by mTOR, a downstream marker 31. The Akt/mTOR signaling regulates the expression of protein Nrf2 in cancer cells, which in turn regulates many biological behaviours 13, 32. Lots of studies have shown that activated Akt could phosphorylate Nrf2, resulting in the release of Nrf2 from KEAP1-NRF2 complex 33. And then Nrf2 transfers to the nucleus, ultimately leading to the transcriptional activation of phase II enzyme/antioxidant genes. Nrf2 is a well-known transcription factor. When Nrf2 is blocked, the growth of cancer is inhibited. Cell proliferation and invasion were immediately affected by activating Akt phosphorylation in our research. In this study, Nrf2 was markedly down-regulated along with down-regulation of p-Akt and p-mTOR. Following this we used Akt agonist SC79 for the reverse experiment. As we expected, the expression of p-Akt and Nrf2 was increased in SC79 treatment group, and SC79 treatment reversed the inhibition of knockdown of CSF2 on the proliferation and invasion of cells. Our findings confirmed that CSF2 knockdown reduces Nrf2 expression and inhibits Akt/mTOR pathways. Hence, these results imply that the Akt pathway may be involved in the progression of BCa through CSF2.

## Conclusion

We described the molecular mechanism of CSF2 in BCa process, in which CSF2 was high expression. We revealed the expression of CSF2 could attenuate BCa cell progression for the first time by regulating cell growth. Knockdown of it could obviously increase cancer cell apoptosis and inhibit cancer cell EMT process. Akt/mTOR signaling pathways also regulate the Nrf2 transcription factor via the underline mechanism. As a result of this mechanism, CSF2 may serve as a valuable diagnostic marker in BCa and as a therapeutic target.

## Figures and Tables

**Figure 8:**
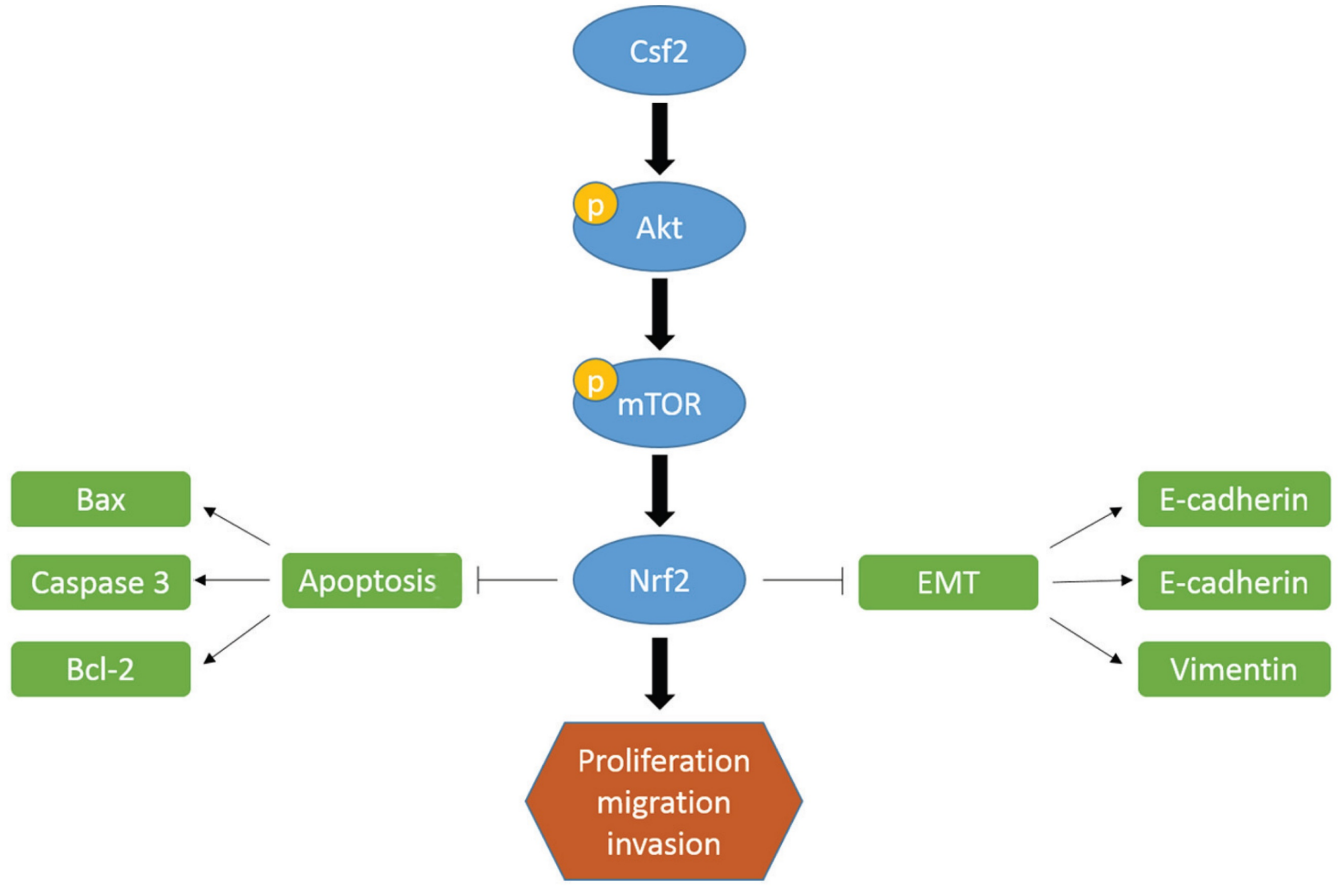
Based on the above results, Csf2 may prompt BCa progression through by regulating Nrf2 via the Akt pathway.

**Table 1:** Correlation between CSF2 expression and clinical features of patients.

Variable	Groups	Total	CSF2 expression		p value
			Low	High	
Gender	Male	41	6	35	0.772
	Female	11	2	9	
Age (years)	≥65	28	5	23	0.593
	<65	24	3	21	
BMI	≥24	27	4	23	0.906
	<24	25	4	21	
Diabetes status	No	39	7	32	0.374
	Yes	13	1	12	
Tumor size (cm)	≥3	30	4	26	0.632
	<3	22	4	18	
Tumor grade	PUNLMP or low grade	10	5	5	0.001
	High grade	42	3	39	
Tumor stage	Ta, T1	13	5	8	0.001
	T2-T4	39	3	36	
Lymphnodes	Negative	44	6	38	0.412
	Positive	8	2	6	
Distant metastasis	Negative	50	8	42	0.539
	Positive	2	0	2	

## Abbreviations

BCa: Bladder Cancer
CSF2: Colony-stimulating factor 2
TCGA: the Cancer Genome Atlas
BCG: Bacteria Calmette Guerin
DC: dendritic cells
GM-CSFR: ligand-specific subunit receptor
IHC: Immunohistochemistry
H_2_O_2_: hydrogen peroxide
BCA: bicinchoninic acid
PFA: paraformaldehyde
PBS: phosphate buffer solution
SPF: specific pathogen-free
